# Supplementary material for: Antibody Generation and Rapid Immunochromatography Using Time-Resolved Fluorescence Microspheres for Propiconazole: Fungicide Abused as Growth Regulator in Vegetable
Source: Foods. 2022 Jan 24;11(3):324. doi: 10.3390/foods11030324 (PMC8834100; doi:10.3390/foods11030324)
Supplement: Supplementary file 1 [file foods-11-00324-s001.zip › foods-1486116-supplementary.pdf]

Supporting information

Antibody generation and rapid immunochromatography using time-resolved fluorescence microspheres for propiconazole: fungicide abused as growth regulator in vegetable

Bo Chen [1], Xing Shen [1], Zhaodong Li [2], Jin Wang [1], Xiangmei Li [1], Zhenlin Xu [1], Yudong Shen [1], Yi Lei [3], Xinan Huang [4], Xu Wang [5], Hongtao Lei [1,*]

1 Guangdong Province Key Laboratory of Food Quality and Safety, College of Food Science, South China Agricultural University, Guangzhou 510642, China

2 College of materials and energy, South China Agricultural University, Guangzhou 510642, China

3 Guangdong Institute of Food Inspection, Zengcha Road, Guangzhou 510435, China

4 Tropical Medicine Institute and South China Chinese Medicine Collaborative Innovation Center, Guangzhou University of Chinese Medicine, Guangzhou 510405, China

5 Institute of Quality Standard and Monitoring Technology for Agro-products of Guangdong Academy of Agricultural Sciences, Guangzhou 510405, China

* Corresponding author. Hongtao Lei (hongtao@scau.edu.cn), Tel: +8620-8528 3925. Fax: +8620-8528 0270.

* Corresponding author.

Hongtao Lei (hongtao@scau.edu.cn), Tel: +8620-8528 3448. Fax: +8620-8528 0270.

This file includes:

Supplementary Figures S1-S6

Supplementary Figures

华南农业大学
South China Agricultural University

Results of ethical review of animal experiments

No：2020e009

Experiment Item	Research on the Key Technology of Multidimensional Inspection of the Authenticity of Important Food			
Application number	2020E009			
Comments on conservation of experimental animals	All the experimental mice used in this experiment came from an experimental animal center legal license. The type, quantity, and grouping of mice were conformed to the 3R principle.			
Comments on ethical and moral	This experiment was carried out in a laboratory with a license for experiment animals, which was confirmed to the welfare principle.			
Comments on ethical and moral	The animals were euthanized after the experiment.			
Comments on comprehensive scientific evaluation	This experimental study has scientific significance.			
Time of experiment animal type and quantity	Date: 2020-09-01 to 2012-12-01.<br>Experimental animal: CV New Zealand white rabbit.<br>Quantity: 10 female and 10 male			
Comments of the ethical reviewer	Agree			
	Reviewer	Wei Huang	Review Date	2020-08-16
Comments of the ethical reviewer	Agree			
	Reviewer	Rangcai Yu	Review Date	2020-08-16
Final comments of the director (or deputy director)	Agree			
	Review	Zhonghua Liu	Review Date	2020-08-16

Experimental Animal Ethics Committee of
South China Agricultural University

Date: 2020-08-16

Figure S1. Ethical review of animal experiments.

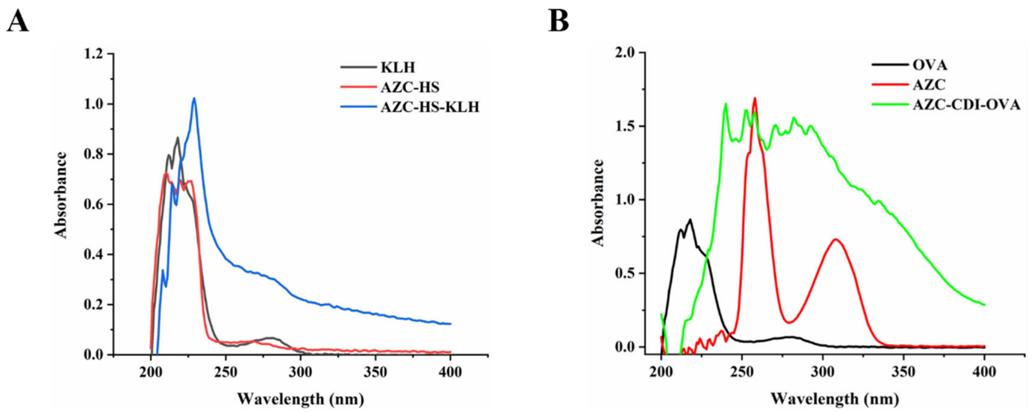

Figure S2. The result of hapten-carrier conjugation. (A) The UV-VIS spectroscopy of AZC-HS, KLH, and conjugates. (B) The UV-VIS spectroscopy of AZC, OVA, and conjugates. UV-VIS, Ultraviolet-visible spectroscopy. KLH, Keyhole limpet hemocyanin. OVA, Albumin from chicken egg white. CDI, N, N'-Carbonyldiimidazole.

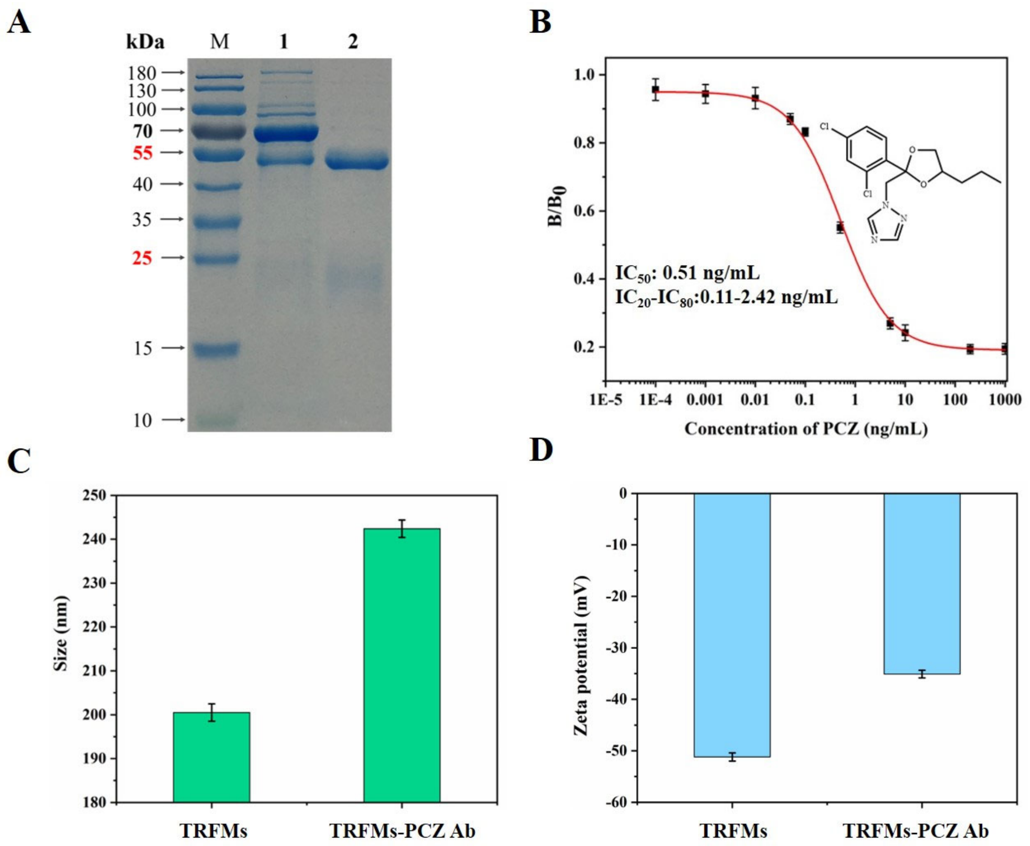

Figure S3. Characterization of PCZ Ab and TRFMs-PCZ Ab conjugation. (A) The result of SDS-PAGE. Lane M, standard protein markers. Lane 1, Before purification. Lane 2, After purification. (B) Standard curve of antibody. (C) The average particle size of TRFMs and TRFMs-PCZ Ab. (D) The zeta potential of TRFMs and TRFMs-PCZ Ab.

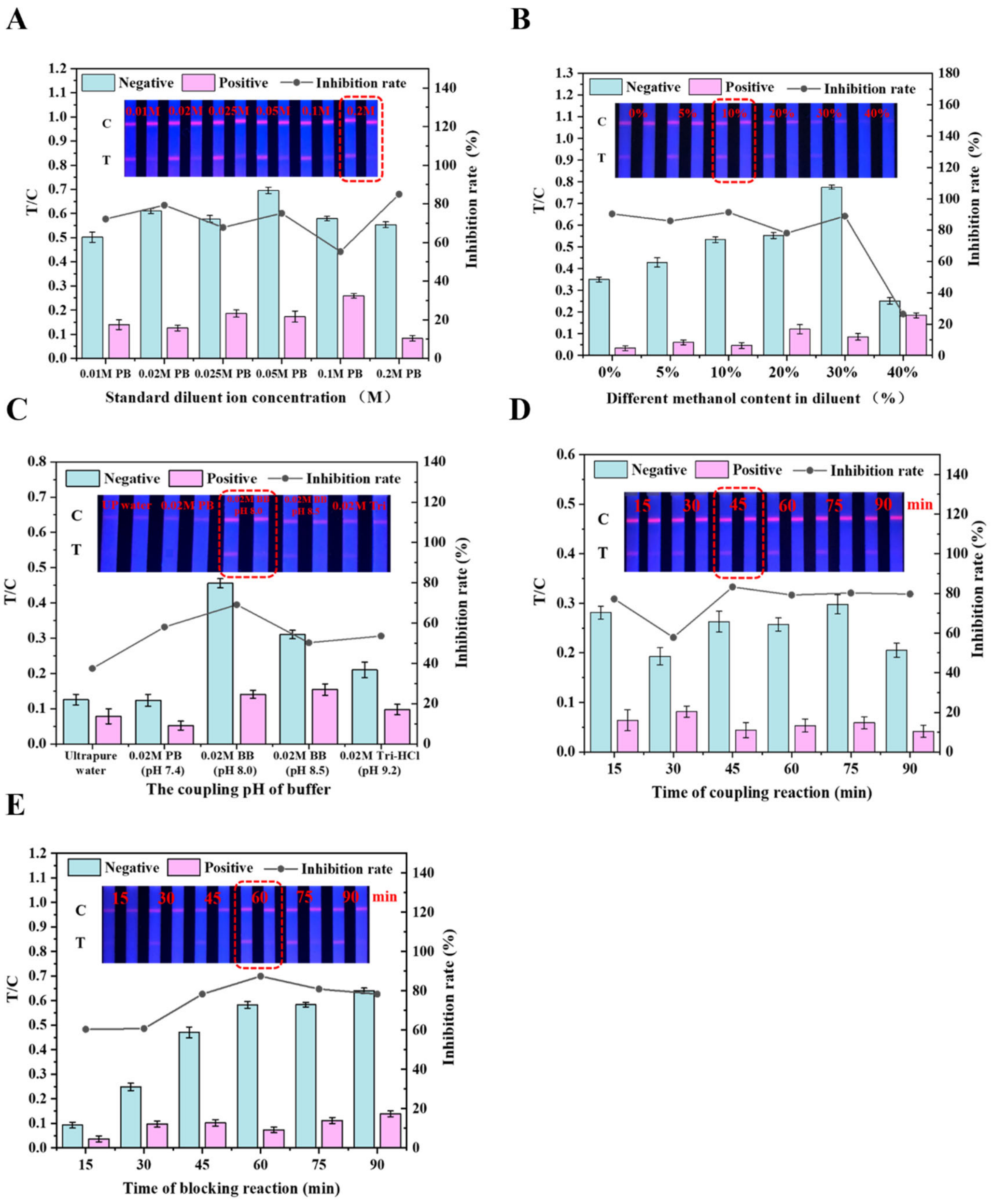

Figure S4. Optimization of other working conditions. All the optimize conditions were evaluated by the negative (0 ng/mL) and positive (100 ng/mL). The values of T/C below was calculated from the pictures above by FIC-Q1 fluorescence reader and the screening criteria combined the T/C value, where B and B_0 were the ratio of T/C values with and without propiconazole in the sample solutions. Inhibition rate is equal to $1-B_0/B$. (A) Standard diluent ion concentration, (B) Methanol content in diluent, (C) Coupling pH, (D) Time of coupling reaction, (E) Blocking time.

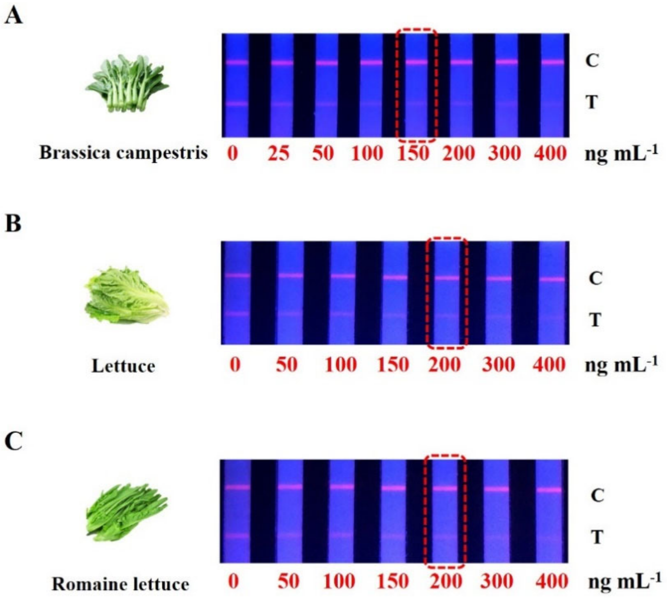

Figure S5. Detection results of propiconazole in (A) brassica campestris, (B) lettuce, and (C) romaine lettuce samples by TRFM-LFIA. Red rectangular box represents the vLOD concentrations of propiconazole by TRFMs-LFIA.

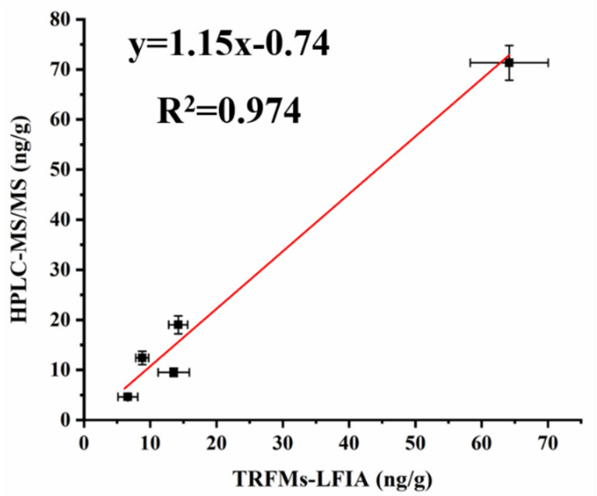

Figure S6. The correlation diagram of blind sample detection results of the TRFMs-LFIA and HPLC-MS/MS (n=3)
